# Formulation of Gluten‐Free Biscuits Based on Tiger Nut, Dana, and Mixture of Avocado and Margarine: Nutritional Composition and Glycemic Index of Optimal Sample

**DOI:** 10.1002/fsn3.71852

**Published:** 2026-05-03

**Authors:** Ghislain Maffo Tazoho, Booh'boo Tchassi, Donald Sévérin Dangang Bossi, Stephano Tambo Tene, Hilaire Macaire Womeni, Inocent Gouado

**Affiliations:** ^1^ Department of Biochemistry, Faculty of Science University of Dschang Dschang Cameroon; ^2^ Department of Biochemistry, Faculty of Science University of Douala Douala Cameroon

**Keywords:** glycemic index, local food plants, mixture design, nutritional properties, optimal gluten‐free biscuit

## Abstract

The aim of this work was to develop a low‐glycemic‐index, gluten‐free biscuit by completely replacing wheat flour with flours from three local food plants, namely dana ( *Dioscorea bulbifera* ), tiger nut ( *Cyperus esculentus* ), and a fatty composition consisting of a 50/50 blend of avocado ( *Persea americana* ) and margarine. Using an extreme summit mixture design, nine formulation trials (M1 to M9) were conducted on which the nutritional, functional, and antinutritional properties were assessed. The same design was used to define the optimal formulation according to nutritional composition and sensory appeal. This optimal formulation was characterized as previously described and its glycemic load was also determined using standardized protocols. These analyses showed that the nine mixtures (M1 to M9) obtained had fiber, starch, and reducing sugar content ranging from 10.4% to 14.3%, 13.32% to 29.49%, and 1.26% to 2.98%, respectively. Analysis of the optimal biscuit with a ratio of 58.40/25/16.6 for tiger nut, dana, and fat showed that the fiber content and energy value were 18.95 g/100 g and 449.86 kcal/100 g, respectively. The oil to water absorption capacity ratio was 1.13 ± 0.37 with a pH of 6.49 ± 0.01. The glycemic index of the biscuit was 53.81 ± 5.48 with a glycemic load of 3.4 ± 0.56. In conclusion, the formulated biscuit exhibits favorable nutritional characteristics and a low glycemic index making it a promising future alternative as a snack for people at risk of diabetes.

## Graphical abstract

This study aimed to develop a gluten‐free biscuit with a low glycemic index by completely replacing wheat flour with flours from three local plant foods (tiger nut, dana, and avocado). The methodology began with the production of tiger nut and dana flours and avocado paste followed by the mixing plan and obtention of 09 (nine) formulations. The optimal gluten‐free biscuit with the proportion 58.40% *Cyperus esculentus* (tiger nut), 25% *Dioscorea bulbifera* (dana), and 16.59% fat (made up of 50/50 of avocado ( *Persea americana* ) and margarine) was produced based on the results of nutritional composition and sensory evaluation of nine formulations obtained previously. The results show that dana, tiger nut, and fat used in this study have demonstrated their ability in biscuit production. The optimal biscuit possesses important characteristics in terms of nutritional composition, hedonic properties, and its glycemic index and glycemic load was 53.81 and 3.4 respectively. *In fine*, the optimal biscuit produced in this study, while promoting underutilized local plant materials could thus constitute a promising alternative for most gluten‐free foods that are often rich in carbohydrates but lacking other nutrients (protein, lipids, minerals and fiber) needed for metabolic activities.

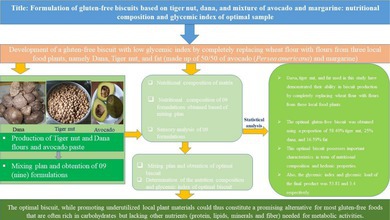

## 1 Introduction

The foods we consume are substances of animal, plant, or mineral origin that satisfy hunger while providing essential nutrients for human health. Global industrialization and shifting trends have led to rapid changes in diets and lifestyles (Njinkoue et al. 2018). This is evident today in the high consumption of baked foods, which are part of people's daily diets. As a result, the prevalence of metabolic diseases such as obesity and diabetes mellitus is rising. Diabetes is now a major global public health problem, with a steadily increasing prevalence, particularly in low‐ and middle‐income countries (International Diabetes Federation 2023). The consumption of baked foods such as bread, cakes, and biscuits has increased significantly due to urbanization, the convenience of ready‐to‐eat foods, and the availability of various products with different flavors and texture characteristics, as well as their high nutritional profile and long shelf life (Chikwendu et al. 2021). Biscuits are widely accepted and enjoyed baked foods around the world. They are the most popular snacks among young people and adults and generally have a high carbohydrate, fat, and energy content but lack sufficient dietary fiber (Eyenga et al. 2021).

Biscuits are generally made from wheat flour, sugar, and fat (Chandana and Navaratne 2015). However, in high quantities, these ingredients are unsuitable for people with obesity and diabetes (Ajay and Pradyuman 2018). In Cameroon, almost all wheat is imported, and it is notable that in the second quarter of 2025, Cameroon imported 250,000 tons of wheat, bringing the total to over 1 million ton annually at a cost of approximately 220 billion CFA francs. Cameroon's dependence on these exporting countries is a major concern for the food security of its population, given that the consumption of wheat‐based flour products causes certain health problems, the most well‐known being gluten intolerance. To overcome these problems, scientists are increasingly interested in formulating baked products from local resources by totally or partially replacing wheat flour (Eyenga et al. 2021; Ndjang et al. 2023; Tambo et al. 2024). Following this logic, Mingle et al. (2017) developed bread made from dana flour and found that using 25% dana flour produced sensory effects comparable to those of bread containing 100% wheat flour. The use of 50% dana flour was deemed acceptable by consumers (Nwosu 2014). In the same vein, Bansod et al. (2020), after exploring the potential of dana flour in baking, concluded that up to 60% substitution of this flour in biscuit formulations was acceptable to consumers compared to wheat flour biscuits.

Several studies have been conducted on the formulation of bakery products based mainly on tiger nuts or dana ( *Dioscorea bulbifera* ). However, to the best of our knowledge, little or no scientific work has been on the formulation of biscuits using a combination of tiger nuts ( *Cyperus esculentus* ), and dana ( *Dioscorea bulbifera* ), nor the utilization of avocado ( *Persea americana* ), hence the interest of this study. *Cyperus esculentus* (tiger nut) grains are rich in fat, carbohydrates, and dietary fiber (Zhang et al. 2022), and their incorporation into formulations or diets improves the nutritional profile by increasing the protein, fiber, mineral, and vitamin content (AlJuhaimi et al. 2024). The fruits of *Persea americana* are excellent sources of fiber, monounsaturated fatty acids, antioxidants (polyphenols), vitamins, and minerals such as folic acid, pantothenic acid, copper, potassium, vitamin K, and vitamin B_6_ (Ford et al. 2023; Siol and Sadowska 2023). *Dioscorea bulbifera* is known for its antioxidant, antibacterial, analgesic, hypoglycemic, anti‐inflammatory, and lipid‐lowering properties due to the bioactive compounds it contains, such as polyphenols and flavonoids (Chinko et al. 2020; Padhan and Panda 2020).

The incorporation of functional foods such as Dana with proven biological properties into the diets of those suffering from diabetes, obesity, hypertension, and other metabolic diseases is of increasing interest from a health perspective, as the development of these conditions poses a real public health problem (Jayedi et al. 2020; Dragomir et al. 2023). Increasingly, high‐fiber and cost‐effective diets (based on local foods) are becoming more popular in the fight against diabetes and other metabolic disorders through the control of blood sugar and cholesterol levels. Numerous studies have been conducted with a view to reducing the glycemic load of foods and their effect on consumer health by combining foods such as legumes (soybeans, cowpeas, and beans) and starchy foods (corn, yams, and bananas) (Reynolds et al. 2020; Waddell and Orfila 2023; Njapndounke et al. 2023; Saha et al. 2025). Nevertheless, research continues to focus on new matrices such as *Cyperus esculentus, Dioscorea bulbifera*, and *Persea americana* .

Given this information, we ask the question whether using a mixture design of avocado, tiger nut, and dana flours would be possible to produce a gluten‐free biscuit with a low glycemic index and reducing‐sugar content, and a higher content of fiber, proteins, and antioxidant compounds, while remaining appealing to consumers? Therefore, the objective of this study was to formulate a gluten‐free biscuit with a low glycemic index and enhanced nutritional properties by completely replacing wheat flour with flours from *Dioscorea bulbifera* and *Cyperus esculentus* and paste from *Persea americana* .

## 2 Materials and Methods

### 2.1 Materials

Yellow tiger nut ( *Cyperus esculentus* ) seeds were collected from farmers in the Mayo‐Louti Division (Guider Subdivision), while white potato ( *Dioscorea bulbifera* ) tubers and avocado ( *Persea americana* ) fruits were harvested from farmers in Dschang (Menoua Division). These matrices were collected between March and April 2025 (during the dry season) and then transported to the Research Unit of Biochemistry, Medicinal Plants, Food Science and Nutrition at the Department of Biochemistry, Faculty of Sciences, University of Dschang, for processing.

The chemicals (Gallic acid, catechin, trichloroacetic acid, ammonium thiocyanate, ferrous chloride, boric acid, potassium permanganate, vanillin, sodium and potassium hydroxides, hexane, methanol, hydrochloric and sulfuric acids) were obtained from Sigma‐Aldrich (HPLC grade or 99% purity) and were purchased from a local seller in Bafoussam (West Region of Cameroon).

### 2.2 Methods

#### 2.2.1 Production of Tiger Nut and Dana Flour

The tiger nuts were sorted, washed twice with running water and drained. After draining, they were dried at 45°C for 72 h in an oven (Venticell, Local brand, Douala, Cameroon). The dried grains were then ground using a blender mill (POLYMIX KINETICA, Japan) and sieved using a sieve with a pore diameter of 200 μm. Finally, the resulting flour was packaged in plastic bags and stored at room temperature, away from light in a desiccator. The dana tubers were washed with tap water and boiled for 45 min at 95°C using an electric induction cooker (Prestige PIC 6.1 V3, India). They were then peeled and cut into 1 cm squares using a manual chopper (local brand, Dschang, Cameroon). The pieces obtained were dried at 45°C for 72 h in an oven. The dried pieces were then ground using a SCATH food processor (Magimix Food Pro 4200 XL, Australia) and sieved through a 200 μm mesh. The resulting flour was packaged and stored as previously described. The flours were subsequently used for techno‐functional, and nutritional characterization as well as for biscuit formulation.

#### 2.2.2 Production of Avocado Paste

Avocados were washed, peeled, pitted, and the pulp was collected. The pulp was cut into small pieces and ground using a blender (POLYMIX KINETICA, Japan) to obtain a paste, which was immediately characterized nutritionally and used in the biscuit formulation.

#### 2.2.3 Production of Gluten‐Free Biscuits

##### 2.2.3.1 Experimental Design and Formulation of Different Gluten‐Free Biscuits

The extreme summit mixture design was used in this study, and the factors chosen were tiger nut flour (F1), Dana flour (F2), and fat (F3). The experimental ranges chosen were 40–60 g, 25–50 g, and 16–25 g for F1, F2, and F3, respectively and this was based on preliminary tests and literature review. This design yielded nine (9) formulations, the compositions of which are presented in Table 1. Preliminary tests indicated that to achieve optimal nutritional properties (high fiber, protein, and lipid content), a 1:1 ratio of margarine and avocado was required as the fat source. To prepare the biscuit, GINA brand sugar (8.59 g) and eggs (30 g) were mixed using a RAF brand pastry mixer (R.6671, 1400 W, Zheijang, China) at maximum speed for 2 min. The fat (50% Jadida margarine and 50% avocado pulp) was then added and blended. After mixing for 3 min, the tiger nut and dana flours were incorporated and the dough was kneaded, rolled out, and molded before being baked in an OSCAR oven (USA) at 175°C for 30 min. The baking conditions were strictly controlled, using the same mold to produce all the samples, and the oven was placed in a well‐ventilated laboratory to ensure proper air circulation resulting in biscuits with an average thickness of 0.5 cm. Each biscuit was produced in triplicate (biological replicate) and analyzed in triplicate. The average value for each parameter of each biscuit was used as a replicate to calculate the final average.

**TABLE 1:** Different proportions of each matrix per mixture.

Mixtures	Tiger nut (F1)	Dana (F2)	Fat (F3)
M1	40	44	16
M2	40	34.19	25.81
M3	59	25	16
M4	49.19	25	25.81
M5	47.04	32.04	20.90
M6	43.52	38.02	18.45
M7	43.52	33.12	23.35
M8	53.02	28.52	18.45
M9	48.12	28.52	23.35

##### 2.2.3.2 Selection of the Optimal Gluten‐Free Biscuit

After formulation and characterization, eight parameters were used to determine the optimal mixture using Minitab 18.1 software: lipid content (minimized), carbohydrates (maximized), fiber (maximized), phenols (maximized), flavonoids (maximized), and reducing sugars (minimized), as well as taste (maximized) and overall acceptability (maximized) were used to determine the optimal mixture using Minitab 18.1 software. The mathematical Equations ((1), (2), (3), (4), (5), (6), (7), (8)) for these parameters were validated by determining their coefficient of determination (R^2^), the mean absolute average deviation (MAAD), the bias factor (Bf), and the desirability (D), which should be greater than 0.75, close to 0, between 0.75–1.25, and greater than 0.50, respectively. At the end, the optimal biscuit had the following proportions 58.40/25/16.6 respectively for tiger nut, dana and fat.

(1) $$\begin{array}{cc} \text{Lipids}\;=\; & 0.23{\text{6F}}_{1}+0.04{\text{5F}}_{2}+0.308-0.00{\text{1F}}_{1}F_{2}+0.00{\text{3F}}_{1}F_{3}\\ & +0.00{\text{3F}}_{2}F_{3}\;\left(R^{2}=1;\text{MAAD}=0;\mathrm{Bf}=0.99;D=0.86\right) \end{array}$$

(2) $$\begin{array}{cc} \text{Carbohydrates}\;=\; & 0.69{\text{9F}}_{1}+0.0906F_{2}+0.00{\text{4F}}_{3}-F_{1}F_{2}+0.0002F_{1}F_{3}\\ & -0.0002F_{2}F_{3}\;\left(R^{2}=1;\text{MAAD}=0;\mathrm{Bf}=1;D=0.85\right) \end{array}$$

(3) $$\begin{array}{cc} \text{Fibers}\;=\; & 0.22{\text{6F}}_{1}+0.05{\text{7F}}_{2}-0.26{\text{3F}}_{3}-0.00{\text{1F}}_{1}F_{2}+0.00{\text{4F}}_{1}F_{3}\\ & +0.00{\text{3F}}_{2}F_{3}\;\left(R^{2}=1;\text{MAAD}=0;\mathrm{Bf}=1;D=0.97\right) \end{array}$$

(4) $$\begin{array}{cc} \text{Taste}\;=\; & 0.10{\text{6F}}_{1}-0.05{\text{3F}}_{2}+0.50{\text{8F}}_{3}+0.00{\text{2F}}_{1}F_{2-}0.00{\text{9F}}_{1}F_{3}\\ & -0.00{\text{3F}}_{2}F_{3}\;\;\left(R^{2}=1;\text{MAAD}=0.02;\mathrm{Bf}=1;D=0.98\right) \end{array}$$

(5) $$\begin{array}{cc} \text{General acceptability}\;=\; & 0.19{\text{2F}}_{1}+0.11{\text{5F}}_{2}-0.03{\text{9F}}_{3}\\ & -0.00{\text{4F}}_{1}F_{2-}0.00{\text{2F}}_{1}F_{3}+0.00{\text{5F}}_{2}F_{3}\;\\ & \left(R^{2}=1;\text{MAAD}=0.016;\mathrm{Bf}=1;D=0.94\right) \end{array}$$

(6) $$\begin{array}{cc} \text{Phenols content}\;= & \;0.74{\text{3F}}_{1}-2.44{\text{9F}}_{2}+22.45{\text{1F}}_{3}+0.09{\text{2F}}_{1}F_{2}\\ & -0.36{\text{5F}}_{1}F_{3-}0.28{\text{9F}}_{2}F_{3}\;\\ & \left(R^{2}=1;\text{MAAD}=0.40;\mathrm{Bf}=1.03;D=1\right) \end{array}$$

(7) $$\begin{array}{cc} \text{Flavonoids content}\;=\; & 1.59{\text{9F}}_{1}+4.9024F_{2}-28.55{\text{1F}}_{3}-0.21{\text{8F}}_{1}F_{2}\\ & +0.4264F_{1}F_{3}+0.4178F_{2}F_{3}\;\\ & \left(R^{2}=0.87;\text{MAAD}=0.13;\mathrm{Bf}=0.99;D=1\right) \end{array}$$

(8) $$\begin{array}{cc} \text{Reducing sugars}\;=\; & 0.18{\text{2F}}_{1}+0.41{\text{7F}}_{2}+0.72{\text{4F}}_{3}-0.01{\text{0F}}_{1}F_{2}\\ & -0.01{\text{2F}}_{1}F_{3}-0.01{\text{2F}}_{2}F_{3}\;\\ & \left(R^{2}=0.87;\text{MAAD}=0.10;\mathrm{Bf}=1;D=0.84\right) \end{array}$$

*With F1: linear effect of tiger nut flour; F2: linear effect of dana flour; F3: linear effect of fat (mixture of 50% avocado and 50% margarine); F1F2; F1F3; F2F3: interactions between the different factors*.

#### 2.2.4 Nutritional and Techno‐Functional Characterization of Plant Materials and Formulated Biscuits

##### 2.2.4.1 Macronutrient Content

The macronutrient composition was determined according to the AOAC (2005) method. For water content, a small amount of each sample was placed in porcelain crucibles and dried at 105°C in a Venticell (MM‐group) ventilated oven. Lipid content was determined by solubilization in a nonpolar organic solvent (hexane), while protein content was determined using the standard Kjeldahl method. The crude fiber content was determined using Scharrer's reagent, and in a muffle furnace, the sample was calcined at 450°C for 3 h, and the fiber content was calculated by mass difference. Reducing sugar content was determined via the (AACC American Association of Cereal Chemists (1990)) method using 3,5‐dinitrosalicylic acid (DNSA) after water extraction and precipitation with 2% zinc acetate and 10.6% ferrocyanide. Total digestible carbohydrate content was calculated by difference following the method of Zubair et al. (2016) using Equation (9).

(9) $$\begin{array}{cc} \%\text{Carbohydrates}\;=\; & 100–(\%\text{Proteins}+\%\text{Lipids}\\ & +\%\mathrm{Ash}+\text{Fiber}+\text{Moisture}) \end{array}$$

(10) $$\begin{array}{c} \text{Energy was calculated using the formula in equation}\;10\;\\ \;\mathrm{as}\;\text{described}\;\mathrm{by}\;\mathrm{WHO}/\mathrm{FAO}\;\left(2003\right).\;\text{Energy}\;\left(\text{kcal}/10\text{0g}\right)\\ \;=4\times \%\text{total carbohydrates}+4\times \%\text{crude proteins}+9\times \%\text{lipids} \end{array}$$

##### 2.2.4.2 Mineral Determination

Mineral content (phosphorus, iron, copper, manganese, potassium, sodium, calcium, and magnesium) was measured according to the method described by Pauwels et al. (1992). After sample ashing and digestion with an HNO^3^/HCl mixture, each mineral was quantified using specific standard solutions. Absorbance was measured using a Perkin Elmer HGA 700 flame spectrophotometer (USA) at the following wavelengths: 420 nm for phosphorus, 510 nm for iron, 768 nm for potassium, and 589 nm for sodium. Calcium and magnesium contents were calculated using specific equations.

##### 2.2.4.3 Techno‐Functional Properties of Plant Materials and Formulated Biscuits

The swelling rate (SR), water absorption capacity (WAC), and oil absorption capacity (OAC) were determined according to the method described by Okezie and Bello (1988). Briefly, 10% (w/v) flour dispersions (in oil or water) were prepared and heated to 30°C in a calibrated water bath. The mixtures were stirred at 80 rpm for 30 min, then centrifuged at 4500 rpm for 15 min. The supernatant from each tube was removed and its volume recorded (V_2_), while the resulting paste was weighed. The SR, WAC, and OAC were calculated using the respective Equations (11) and (12).

(11) $$\text{Swelling rate}\;\left(\%\right)=\frac{M1-M0}{M1}\times 100$$

(12) $$\mathrm{WAC}/\mathrm{OAC}\;\left(\%\right)=\frac{\left(V_{0}-V_{1}\right)}{V_{0}}\times 100$$

where M_1_ is the mass of the base or mass after water absorption (g); M_0_ is the mass of the test sample (g); V_0_ is the initial volume (mL) and V_1_ is the volume after absorption of oil or water (mL); WAC/OAC = water or oil absorption capacity.

The method described by Okaka et al. (1991) was used to determine the bulk density using the formula in Equation (13):

(13) $$\text{Bulk density}\;\left(g/\mathrm{mL}\right)=\frac{M1-M0}{V}$$

where M_0_ is the mass of the empty crucible, V the volume of flour, and M_1_ the mass of the crucible filled with flour.

The pH was measured according to the AOAC (2005) method. A 10% (w/v) suspension prepared with distilled water was stirred using a Barnstead/Thermolyne vortex mixer (model LS3, Chennai, India) for 30 min and then centrifuged at 4500 rpm for 15 min using a Heraeus refrigerated centrifuge. The pH of the supernatant was measured using a calibrated pH meter at 25.03°C ± 0.22°C.

##### 2.2.4.4 Determination of Antinutritional Compounds in the Samples

###### 2.2.4.4.1 Determination of Phytate Content

The phytate content was determined using the AOAC (2005) method. Briefly, 2.0 g of sample and 100 mL of 2% HCl were placed in a 250 mL Erlenmeyer flask and allowed to stand for 12 h. The solution was then filtered through a fine‐mesh filter. Subsequently, 50 mL of the filtrate, 107 mL of distilled water, and 10 mL of 0.3% ammonium thiocyanate were mixed. The resulting solution was titrated with a standard solution FeCl_3_.6H_2_O (containing 0.00195 g iron per mL) until a persistent yellow‐orange endpoint was reached and maintained for 5 min. The phytate content was expressed in mg/100 g.

###### 2.2.4.4.2 Determination of Oxalate Content

The oxalate content was determined according to the method of Day and Underwood (1986). One gram of each sample was weighed and added to 75 mL of 1.5 M. After 8 h of extraction, the mixture was filtered, and a 25 mL aliquot of the filtrate was titrated at 90°C with 0.1 M KMnO_4_ solution until a persistent pink color was maintained for 30 s. The oxalate content, expressed in mg/100 g, was calculated using Equation (14):

(14) $$0.1\;\mathrm{mL}\;{\text{KMnO}}_{4}=0.00450\;g\;\text{Oxalate}$$

###### 2.2.4.4.3 Determination of Tannin Content

The condensed tannin content was assessed using the method described by Luzardo‐Ocampo et al. (2017). Briefly, 1 mL of each extract (2% in MeOH/HCl 1%) was mixed with 5 mL of a reagent solution consisting of 0.5 g of vanillin and 8 mL of hydrochloric acid in 100 mL of distilled water. The mixture was incubated at 30°C for 20 min, and the absorbance was read at 500 nm against a blank.

###### 2.2.4.4.4 Determination of Saponin Content

The saponin content was determined using the method described by Kozioł (1992). Briefly, 0.5 g of the sample and 5 mL of distilled water were mixed in a test tube. The mixture was shaken vigorously for 30 s, and the height of the resulting foam was measured using a ruler graduated to the nearest 0.1 cm. The concentration of saponins was calculated using Equation (15):

(15) $$\text{Saponins}\;\left(\mathrm{mg}/100\;g\right)=\frac{\left(0,432\times \text{foam heignt in}\;\mathrm{cm}\right)+0,008}{\text{sample weight}\;\left(g\right)}$$

###### 2.2.4.4.5 Determination of Cyanogenic Glycoside Content

The cyanogenic glycoside content was determined using the alkaline picrate paper method described by Nwokoro et al. (2009). A sample extract was prepared by mixing 100 mg of powder with 1 mL of phosphate buffer (0.2 M; pH 8). A piece of picrate paper was attached to a circular stopper and positioned to suspend in the headspace above the liquid phase. The bottle was immediately sealed with a screw cap; after 24 h of incubation at 30°C, the paper was removed, immersed in 5 mL of distilled water, and gently shaken for 30 min at room temperature. The solubilized paper was removed, and the tube was placed in a boiling water bath (Model TT 9052, Techmel & Techmel, USA) for 5 min. The solution was centrifuged at 4500 rpm for 5 min, and the absorbance was measured at 510 nm against a blank. Results were expressed as mg HCN/100 g using the calibration curve equation: y = 17.764×, R^2^ = 0.998.

###### 2.2.4.4.6 Determination of Total Phenols and Flavonoids

The aqueous extract was prepared by mixing 100 g of each flour with 250 mL of distilled water (2:5 w/v) in flat‐bottomed flasks. The mixture was manually stirred twice daily for 48 h at 26°C and then filtered through Whatman No. 4 filter paper. The resulting filtrates were dried at 45°C for 48 h in a ventilated drying oven (Venticell, MM‐Group, Douala, Cameroon).

Total phenolic content was determined by using the methods of Gao et al. (2000) using with a 2 mg/mL of the dry extract. Specifically, 10 μL of each sample, 200 μL of Folin–Ciocalteu reagent (1/10; v/v), and 1.39 mL of distilled water were mixed together into a test tube followed by 400 μL of sodium carbonate (20%) after 3 min. Using a water bath (MODEL TT 9052, Techmel & Techmel USA) at 40°C, the whole tube was incubated for 20 min and then absorbance was read against a blank at 760 nm using a UV visible spectrophotometer (Genesys 50, Thermo scientific, USA). Concomitantly, a freshly prepared aqueous solution of gallic acid (0.2 g/L) was used for calibration and the results were expressed as mg gallic acid equivalent/g extract (mg GAE/g) using the calibration curve equation y = 0.042×, R^2^ = 0.993.

Flavonoid content was determined following the protocol of Bahorun et al. (2004) using a 2 mg/mL extract solution. Specifically, 100 μL extract, 30 μL of 5% sodium nitrite solution, and 1.4 mL distilled water were mixed. After 5 min, 240 μL of distilled water, 200 μL of 10% aluminum trichloride, and 200 μL of 10% sodium hydroxide (NaOH) were added. The mixture was vortexed for 5 min, and the absorbance was measured at 510 nm against a blank. Results were expressed as mg catechin equivalent per gram of extract (mg CE/g) using calibration curve equation y = 0.0867×, R^2^ = 0.97.

#### 2.2.5 Sensory Analysis of the Formulated Biscuits

Based on the biscuit formulations presented in Table 1, a sensory analysis was performed on the nine (9) formulated biscuits following the protocol of Stone et al. (2012). This study was granted Ethical Clearance No. 495/28/05/CE/CRERSH‐OU/VP by the Regional Ethics Committee for Human Health Research in the West Region of Cameroon (CRERSH‐West). A hedonic test was conducted with a panel of 61 volunteers (37 males and 24 females) aged 18 to 44. The panelists were recruited among teachers, students, and staff on the university campus. All participants were non‐smokers, in good health, and had no allergies to the ingredients used. Participants were informed of the study's purpose and the evaluation procedures. Each panelist was seated at an evaluation station and provided with water and the nine (9) coded biscuits. A scorecard was used to evaluate each sample across six (6) sensory parameters: taste, aroma, appearance, crispness, texture, and overall acceptability. Ratings were based on a nine‐point hedonic scale ranging from 1 (dislike extremely) to 9 (like extremely). Samples were labeled M1 through M9, and evaluators were permitted to test them in any order. Between samples, each evaluator rinsed their mouth with water and rested for 5 min to ensure that their taste buds could accurately appreciate the subsequent biscuits.

#### 2.2.6 Evaluation of Glycemic Load (GL) and Glucose Response of the Optimal Gluten‐Free Biscuit

In accordance with the ethical clearance number provided above, the Regional Ethics Committee for Human Health Research of the West Region (CRERSH‐West) approved the study. The procedure of Brouns et al. (2005) was used for glucose response evaluation. By giving their informed consent, participants agreed to participate in the test. Fifteen (15) healthy human volunteers (9 males and 6 females) aged 19–29 years with an average body mass index of 23.80 ± 0.14 kg/m^2^ (between 18 and 24.44 kg/m^2^), with no family history of diabetes, non‐pregnant and non‐diabetic were used. Furthermore, participants were non‐smokers, not taking medications, and in good health. They were advised to avoid vigorous activity and caffeine‐containing drinks for 24 h before the test and to perform an overnight fast of 10–12 h. On the first day, participants were given a reference food (50 g of extra‐pure dextrose dissolved in 150 mL of water). Blood glucose was measured from capillary blood via finger prick using a glucometer (Spengler JT 100, France) at 30 min intervals for 2 h (2 h). The glucometer was calibrated with the manufacturer's solution before use. After 48 h, a 78.95 g portion of the test food (optimal biscuit), containing 50 g of available digestible carbohydrates, was administered. Blood collection was repeated at 30, 60, 90, and 120 min. Blood glucose curves were constructed, and the incremental area under the curve (IAUC) was determined using the trapezoidal rule. The procedure was repeated twice for each participant to minimize experimental error. The glycemic index (GI) was calculated according to Equation (16), as described by Wolever and Jenkins (1986).

(16) $$\mathrm{GI}=\frac{\text{IAUC of test food}}{\text{IAUC of reference food}}\times 100$$

The glycemic load (GL) was calculated from the obtained glycemic index following Equation (17) as described by Emaleku (2017):

(17) $$\mathrm{GL}=\frac{\mathrm{GI}}{100}\times \text{Available carbohydrate}$$

#### 2.2.7 Statistical Analysis

Minitab software version 18 was used to obtain a mixture design proposing nine trials. Analysis of Variance (ANOVA) was used to determine the influence of each ingredients on the evaluated parameters and when there was significant difference, fisher PLSD test was used to localize the difference at 5% probability. Microsoft Excel software 2016 was used to present the data resulting from three replications as means with standard deviation and the curve was drawn using the same software.

## 3 Results and Discussion

### 3.1 Nutritional Composition of Tiger Nut, Dana, Avocado, and Formulated Gluten‐Free Biscuits

#### 3.1.1 Proximate Content of Tiger Nut, Dana, Avocado, and Formulated Biscuits

Table 2 represents the proximate composition of plant materials and different formulated biscuits. Research on food matrix effects on diet and health has increased the understanding that the health impacts of a food or diet are more than their composition of nutrients. It is therefore very important to know the contribution of nutrients compared to whole foods and food patterns in food formulation (Weaver and Givens 2025). In this study, we undertook the formulation of a gluten free biscuit, and the starting matrices were tiger nut, dana, and avocado. The results show that the protein and lipid contents of tiger nut flour were 3.7% and 23.03%, respectively. These values are comparable to the results of Ban‐koffi et al. (2005), who reported 6.04% for protein and 24.70% for lipids. The lipid content of approximately 1.49% in dana was also close to the value (2.10%) obtained by Ifeanacho et al. (2017). The carbohydrate content of avocado was 0.46%, which was higher compared to the value of 0.2% found by Dreher and Davenport (2013). The fiber content was very high in tiger nuts (22.73%), compared to dana and avocado, which had very low contents of 2.65% and 3.62%, respectively. The differences observed in macronutrients composition could be related to the varieties, growing conditions, and soil type on which the matrices were grown. Indeed, Ejoh et al. (2006) showed that the site and variety have a significant influence on the chemical composition of plants. Kazantzis et al. (2003) showed that irrigation, harvest time, and storage conditions can also influence the biochemical composition of the almonds and the stability of the oil extracted from them.

**TABLE 2:** Proximate composition of tiger nut, dana, avocado, and formulated biscuits.

Sample	Moisture (%)	Ash content (%)	Fiber (%)	Proteins (%)	Lipids (%)	Carbohydrate (%)	Reducing sugar (%)	Energy (kcal/100 g)
Matrix for biscuits formulation								
F1	4.44 ± 0.12^c^	3.02 ± 0.01^a^	22.7 ± 0.10^a^	3.7 ± 0.08^b^	23.03 ± 0.08^b^	43.08 ± 0.15^b^	1.32 ± 0.05^a^	394.39 ± 6.70^a^
F2	9.10 ± 0.23^b^	3.15 ± 0.09^a^	2.65 ± 0.02^c^	5.1 ± 0.04^a^	1.49 ± 0.05^c^	78.55 ± 0.20^a^	0.97 ± 0.11^b^	347.85 ± 0.02^b^
F3	67.65 ± 0.35^a^	2.50 ± 0.71^b^	3.62 ± 0.33^b^	1.3 ± 0.25^c^	24.5 ± 0.35^a^	0.46 ± 0.12^c^	0.10 ± 0.01^c^	227.42 ± 2.00^c^
Formulated biscuits based on mixture design								
M1	12.80 ± 0.20^h^	2.99 ± 0.02^d^	10.5 ± 0.10^g^	3.7 ± 0.28^a^	18.2 ± 0.28^g^	51.81 ± 0.57^a^	2.98 ± 0.18^a^	385.84 ± 0.28^e^
M2	16.10 ± 0.12^a^	2.92 ± 0.03^h^	10.4 ± 0.12^h^	3.2 ± 0.14^c^	23.2 ± 0.14^bc^	44.18 ± 0.14^c^	2.57 ± 0.03^a^	398.32 ± 0.42^b^
M3	11.90 ± 0.10^i^	2.97 ± 0.01^e^	14.3 ± 0.09^a^	3.5 ± 0.35^b^	22.3 ± 0.14^d^	45.03 ± 0.85^b^	1.26 ± 0.10^c^	394.82 ± 0.28^c^
M4	15.70 ± 0.09^b^	2.91 ± 0.10^i^	12.3 ± 0.10^c^	3.1 ± 0.14^c^	25.2 ± 0.14^a^	40.79 ± 0.28^d^	1.76 ± 0.10^bc^	402.36 ± 0.14^a^
M5	13.60 ± 0.50^ef^	3.07 ± 0.03^b^	12 ± 0.08^e^	3.7 ± 0.49^a^	21.1 ± 0.71^e^	46.63 ± 0.42^a^	1.34 ± 0.46^c^	390.32 ± 0.49^d^
M6	13.00 ± 0.20^g^	3.01 ± 0.20^c^	13.1 ± 0.07^b^	3.4 ± 0.42^b^	22.3 ± 0.14^d^	45.2 ± 0.14^b^	1.46 ± 0.05^bc^	395.10 ± 0.57^c^
M7	14.90 ± 0.08^d^	2.94 ± 0.50^g^	12.1 ± 0.10^d^	3.2 ± 0.14^c^	23.7 ± 0.14^b^	43.16 ± 0.28^c^	1.32 ± 0.03^c^	398.74 ± 0.28^b^
M8	13.50 ± 0.20^e^	2.96 ± 0.40^e^	11.2 ± 0.20^f^	3.6 ± 0.28^a^	20.2 ± 0.14^f^	48.54 ± 0.14^b^	1.42 ± 0.25^bc^	390.36 ± 0.14^d^
M9	15.10 ± 0.30^c^	3.23 ± 0.05^a^	11.2 ± 0.09^f^	3.3 ± 0.42^b^	22.7 ± 0.14^cd^	44.47 ± 0.42^c^	1.95 ± 0.41^b^	395.38 ± 0.42^c^
Optimal gluten‐free biscuit								
OB	5.16 ± 0.04	2.29 ± 0.09	18.9 ± 1.08	3.3 ± 0.10	31.02 ± 0.05	39.24 ± 0.24	1.10 ± 0.05	449.86 ± 10.4

*Note:* F1 = Tiger nut flour, F2 = Dana flour, F3 = Avocado paste, M1‐M9 represent the different biscuits obtained from the three starting matrices based on the mixing plan, OB represents the optimal biscuit. The averages (*n* = 3) in the same column for each parameter that do not share the same letters show a significant difference at *p* < 0.05. As for the rounding method, it was to two decimal places considering it was ≥ 5 or < 5.

The different proportions in the starting matrices varied significantly compared to the formulated biscuits. For example, it is clear that the water content of the optimal biscuit was the lowest (5.16%) compared to that obtained on cowpea, banane cochon, and blend flour by Njapndounke et al. (2023) where values were above 8%. This value was also lower than that of the nine formulations. This can be explained primarily by the addition of avocado paste, which is consistent with the findings of Tambo et al. (2024), who reported an increase in moisture content with increasing proportions of coconut flour. The values obtained, with the exception of those for the optimal biscuit, suggest the need for airtight packaging to preserve organoleptic properties and extend shelf life. In the same vein, the lipids content of the optimal biscuit was the highest (31.02%) and the carbohydrate content was the lowest (39.24%) compared to other formulated biscuits (18.20%–25.20% for lipids and 43.16 to 51.81% for carbohydrates). These results were not in line with those of Olaimat et al. (2023), who found values ranging from 11%–24% and 60%–73% for fats and carbohydrates content, respectively, in the gluten‐free corn‐based biscuit supplemented with different proportions of walnuts, peanuts, or their combination. The levels of these two components are provided by dana flour and avocado, respectively. The levels obtained remain below the recommended values for this type of food for biscuits and above the recommended values for lipids (FAO/WHO 2006). The fiber and protein levels increased proportionally with the tiger nut and dana levels, respectively. The fiber content varied between 10.30% (M2) and 14.30% (M3) for the formulations but remained overall lower than that of the optimal formulation (18.90%). The fiber content obtained is very high (greater than the recommended 5%), making these biscuits foods with added biological value. The protein content varied significantly (*p ≤ 0.05*) between formulations, ranging from 3.1% (M4) to 3.7% (M1 and M5). These levels were statistically close to the optimal formulation. These results remain below the recommended value for this type of food, which is 5% and therefore suggests supplementation because none of the raw samples is a source of proteins. These results were close to those obtained by Deepika et al. (2023) with the proteins content varying between 2.78 to 4.25% when developing gluten‐free biscuits by using flours derived from rice, sorghum, and corn in different ratios. It has been shown that low protein in gluten‐free biscuits generally leads to a brittle, fragile, and overly crumbly texture, as proteins are needed to form a cohesive, structured matrix. Without sufficient protein, the biscuits typically exhibit lower expansion, a higher tendency to break, and reduced dough viscosity (Sahagun and Gomez 2018). Ash refers to the availability of minerals in the food. The levels obtained were 2.29% (OB) and 3.23% (M9). The optimal biscuit had the lowest level, which would be the result of a lower proportion of dana and tiger nut. The values obtained remain within the norm for this type of formulation, which is less than 5%. Similarly, the values obtained are higher than those reported by Saha et al. (2025), which were around 1.5% in a hypoglycemic biscuit. The reducing sugar of the optimal biscuit was 1.10% and this was close to the values obtained by Njapndounke et al. (2023). This low content of reducing sugars in these biscuits would be a good indicator of the strategy for managing diabetes, since this helps reduce blood glucose levels, improves insulin resistance, and assists with weight loss. This low reducing sugar content can be explained by the use of small amount of table sugar and the absence of reducing sugars in the ingredients used. Energy released by a particular food is a critical parameter in nutrition. Several chronic diseases such as obesity, diabetes, and cardiovascular disease have been considered to be caused by excess energy intake. All food manufacturers are now required to label the energy of their products to help consumers control their energy intake (Jiang et al. 2014). The energy value of plant materials in this study was 227.42 kcal/100 g for avocado, 347.85 kcal/100 g for dana, and 394.39 kcal/100 g for tiger nut. These values were lower compared to that of optimal biscuit (449.86 kcal/100 g). It is clear that an increase of fat proportion in optimal biscuit led to increase in the energy value of the formulated biscuit. These results were in line with those of Olaimat et al. (2023).

#### 3.1.2 Micronutrient Content of Plant Materials and Formulated Gluten‐Free Biscuits

Table 3 shows the mineral composition of each plant material and formulated biscuits. Regarding mineral content, apart from avocado, which had low levels, the other two matrices (tigernut and dana) showed good levels of Zn, P, Cu, Mn, K, Na, Ca, Mg, and Fe. The formulated biscuits also present a significant amount of minerals compared to the matrices. The most abundant mineral element was potassium, with a value of 7035.2 mg/100 g (dana flour), and the least abundant was copper, with a value of 0.13 mg/100 g for avocado. Despite the fact that mineral content decreased in the biscuits compared to the starting matrices, the values remained high. Inyang and Victoria (2015) obtained similar results by showing that, depending on the treatments applied and the mixtures design approach, the levels of certain minerals may increase or decrease in the final product. Minerals fulfill a wide variety of functions, such as serving as building materials for bones, regulating muscle and nerve function, and maintaining the body's water balance (Kim and Choi 2013). Therefore, their presence in food is essential for healthy metabolism.

**TABLE 3:** Mineral content of tiger nut, dana, avocado, and formulated biscuits.

Samples	Zn	P	Cu	Mn	K	Na	Ca	Mg	Fe
Matrix for biscuit formulation									
F1	52.10 ± 0.90^b^	727.59 ± 0.5^a^	640.12 ± 0.12^a^	237.89 ± 2.40^a^	5380.0 ± 23.5^b^	471.56 ± 1.2^b^	4560 ± 2.30^b^	447.12 ± 1.20^a^	51.32 ± 0.90^c^
F2	84.79 ± 0.20^a^	536.17 ± 0.3^b^	27.43 ± 0.25^b^	101.05 ± 3.60^b^	7035.2 ± 3.56^a^	575.76 ± 2.9^a^	4880 ± 3.10^a^	417.96 ± 1.04^b^	55.01 ± 0.60^b^
F3	2.59 ± 0.01^c^	54.77 ± 2.51^c^	0.13 ± 0.01^c^	0.15 ± 0.05^c^	57.20 ± 3.98^c^	5.05 ± 3.39^c^	8.70 ± 1.10^c^	21.60 ± 1.20^c^	57.20 ± 3.84^a^
Formulated biscuits based on mixture design									
M1	37.30 ± 0.28^a^	241.3 ± 0.28^a^	437.73 ± 0.28^h^	95.17 ± 0.09^g^	3097.3 ± 0.28^a^	253.6 ± 0.28^a^	2148.8 ± 0.14^a^	183.90 ± 0.14^a^	24.20 ± 0.28^a^
M2	29.01 ± 0.28^c^	192.0 ± 0.14^d^	435.06 ± 0.04^i^	95.19 ± 0.11^g^	2408.4 ± 042^d^	197.2 ± 0.28^e^	1671.1 ± 0.14^c^	142.90 ± 0.28^c^	18.80 ± 0.14^c^
M3	21.10 ± 0.14^e^	139.4 ± 0.28^h^	571.71 ± 0.14^a^	140.37 ± 0.31^a^	1760.7 ± 0.49^i^	144.2 ± 0.28^h^	1221.6 ± 0.42^h^	104.50 ± 0.28^f^	13.80 ± 0.14^e^
M4	21.10 ± 0.14^e^	142.8 ± 0.14^g^	530.33 ± 0.01^c^	117.05 ± 0.03^c^	1761.8 ± 0.57^h^	144.3 ± 0.28^h^	1222.6 ± 0.42^g^	104.50 ± 0.28^f^	13.85 ± 0.14^e^
M5	32.30 ± 0.42^b^	210.1 ± 0.14^b^	511.09 ± 0.01^e^	111.95 ± 0.13^e^	2676.9 ± 0.55^c^	219.2 ± 0.58^c^	1857.2 ± 0.14^b^	158.90 ± 0.14^b^	20.90 ± 0.14^b^
M6	32.30 ± 0.28^b^	208.2 ± 0.14^c^	473.59 ± 0.14^f^	103.57 ± 0.30^f^	2721.8 ± 0.14^b^	228.6 ± 0.42^b^	1857.1 ± 0.85^b^	158.90 ± 0.14^b^	21.01 ± 0.14^b^
M7	28.10 ± 0.14^c^	183.1 ± 0.14^e^	472.26 ± 0.01^g^	103.57 ± 0.30^f^	2388.8 ± 014^e^	202.9 ± 0.14^d^	1618.1 ± 0.14^d^	138.40 ± 0.42^d^	18.30 ± 0.28^c^
M8	24.20 ± 0.14^d^	157.7 ± 0.28^f^	572.08 ± 0.03^b^	126.17 ± 0.14^b^	2053.4 ± 0.28^h^	173.9 ± 0.14^g^	1393 ± 0.01^f^	119.20 ± 0.28^e^	15.70 ± 0.49^d^
M9	24.30 ± 0.14^d^	158.5 ± 0.42^f^	519.89 ± 0.14^d^	114.50 ± 0.28^d^	2065.9 ± 0.45^g^	176.4 ± 0.42^f^	1394.1 ± 0.03^e^	119.20 ± 0.28^e^	15.80 ± 0.14^d^
Optimal gluten‐free biscuit									
OB	22.04 ± 0.20	138.6 ± 0.04	570.90 ± 0.02	141.10 ± 0.08	1758.1 ± 0.09	143.5 ± 0.20	1219.0 ± 1.05	105.10 ± 0.50	12.10 ± 0.01

*Note:* F1 = Tiger nut flour, F2 = Dana flour, F3 = Avocado paste, M1‐M9 represent the different biscuits obtained from the three starting matrices based on the mixing plan. OB represents the optimal biscuit. The averages (*n* = 3) in the same column for each parameter that do not share the same letters show a significant difference at *p* < 0.05. Zn = zinc, P = phosphorus, Cu = copper, Mg = magnesium, K = potassium, Na = sodium, Ca = calcium, Mn = manganese, Fe = iron. As for the rounding method, it was to two decimal places considering it was ≥ 5 or < 5.

It should be noted that the Zn, P, Cu, Mn, and Mg contents in the different formulations depend mainly on the proportion of tiger nut, while those of K, Na, and Mg are mainly provided by dana flour. The iron obtained in these biscuits was mainly provided by the avocado paste. Formulation M2 provided the highest proportions of Zn and P among the biscuits, while K, Na, and Ca were more abundant in formulation M1. The values obtained for (P, Ca, Fe, Na, K) are higher than those reported by Saha et al. (2025). Overall, the levels of all minerals comply with the FAO/WHO (2006) standard, which is at least 5 mg/100 g for Zn, 120 to 1600 mg/100 g for Ca, 600 to 6000 mg/100 g for K, and 120 to 1600 mg for P. Given the high values of these different minerals, the consumption of 100 g of these biscuits can significantly contribute to daily mineral intake, therefore providing roughly adequate intake for these minerals.

### 3.2 Antioxidant and Antinutrient Properties of Plant Materials and Formulated Gluten‐Free Biscuits

Table 4 presents the antioxidant and antinutrient content of the initial matrices and formulated biscuits. Antioxidants are essential compounds in the prevention and management of oxidative stress‐related diseases (Rahaman et al. 2023). The results of this study show that the total phenols and flavonoids of formulated biscuits decreased compared to plant materials (tiger nut, dana and avocado). For optimal biscuit, the total phenols and flavonoids content were respectively 13.46 mg GAE/g extract and 8.51 mg CE/g extract compared to that of avocado paste with the highest value of 21.79 mg GAE/g extract and 11.53 mg CE/g extract respectively. This value of total phenol in optimal biscuit is higher compared to that of AllouchTounsi et al. (2025) who obtained a value of 0.34 mg GAE/g DM in optimum biscuit when exploring the synergistic effects of incorporating locally derived and readily available legume flours, specifically faba bean, lentil, and chickpea, in combination with traditional gluten‐free cereal flours (rice and maize) for the development of innovative gluten‐free biscuits. Despite the differences between these two studies, it is important to note that the results could be influenced by the nature of the matrices, which were distinct in each study. Furthermore, this study utilized extracts, whereas Allouch‐Tounsi et al. used powders. Moreover, it has been indicated that polyphenols content in foods and beverages strongly depends on cultivation, technology processes, and transformation (Nardini 2022).

**TABLE 4:** Antioxidant and antinutrient content of tiger nut, dana, avocado, and formulated biscuits.

Samples	Antioxidant compounds		Antinutrient compounds (mg/100 g)				
	Phenols (mg GAE/g)	Flavonoids (mg CE/g)	Saponins	Oxalates	CG	Phytates	Tannins
Matrix for biscuit formulation							
F1	19.23 ± 0.20^b^	11.25 ± 0.12^b^	0.87 ± 0.21^a^	16.87 ± 0.30^b^	19.78 ± 0.10^a^	91.22 ± 0.04^a^	618.95 ± 5.10^a^
F2	15.38 ± 0.40^c^	17.02 ± 0.30^a^	0.27 ± 0.10^c^	34.87 ± 0.10^a^	8.94 ± 0.12^b^	21.36 ± 0.08^b^	389.00 ± 3.02^c^
F3	21.79 ± 0.09^a^	11.53 ± 0.42^b^	0.53 ± 0.31^b^	34.87 ± 0.09^a^	0.00 ± 0.00^c^	9.07 ± 0.02^c^	529.05 ± 4.20^b^
Formulated biscuits based on mixture design							
M1	8.97 ± 0.10^d^	4.66 ± 0.50^d^	0.31 ± 0.02^a^	14.62 ± 0.19^b^	7.45 ± 0.24^e^	91.54 ± 0.32^a^	201.68 ± 1.63^d^
M2	22.43 ± 0.20^a^	6.86 ± 0.32^c^	0.14 ± 0.02^b^	11.25 ± 0.10^d^	5.01 ± 0.27^f^	64.08 ± 0.05^d^	43.62 ± 0.97^h^
M3	15.38 ± 0.09^bc^	7.41 ± 0.40^bc^	0.10 ± 0.01^c^	11.25 ± 0.10^d^	14.36 ± 0.09^a^	85.31 ± 0.60^b^	180.11 ± 1.33^e^
M4	14.74 ± 0.30^bc^	10.16 ± 0.91^a^	0.10 ± 0.01^d^	15.75 ± 0.17^a^	9.01 ± 0.20^c^	69.93 ± 0.66^c^	139.77 ± 0.86^f^
M5	14.10 ± 0.32^c^	3.84 ± 0.70^de^	0.01 ± 0.00^c^	13.5 ± 0.35^c^	8.67 ± 0.23^d^	54.62 ± 0.44^f^	116.32 ± 1.18^g^
M6	3.85 ± 0.70^e^	2.74 ± 0.61^e^	0.10 ± 0.01^c^	9.00 ± 0.21^f^	4.13 ± 0.04^g^	48.46 ± 0.32^g^	310.50 ± 1.05^a^
M7	8.33 ± 0.56^d^	8.78 ± 0.09^ab^	0.10 ± 0.01^c^	9.00 ± 0.21^f^	4.47 ± 0.16^g^	15.11 ± 0.07^i^	179.17 ± 1.29^e^
M8	18.59 ± 0.08^ab^	3.02 ± 0.81^de^	0.10 ± 0.01^c^	10.12 ± 0.12^e^	13.82 ± 0.58^b^	21.12 ± 0.08^h^	251.87 ± 0.79^c^
M9	17.30 ± 0.10^bc^	6.59 ± 0.30^c^	0.10 ± 0.01^c^	11.25 ± 0.10^d^	2.43 ± 0.39^h^	60.21 ± 0.22^e^	262.19 ± 1.27^b^
Optimal gluten‐free biscuit							
OB	13.46 ± 0.90	8.51 ± 0.50	0.18 ± 0.01	12.30 ± 0.01	14.10 ± 0.13	86.87 ± 4.32	181.11 ± 0.35

*Note:* F1 = Tiger nut flour, F2 = Dana flour, F3 = Avocado paste, M1‐M9 represent the different biscuits obtained from the three starting matrices based on the mixing plan. OB represent the optimal biscuit. CG = cyanogenic glycosides. The averages (*n* = 3) in the same column for each parameter that do not share the same letters show a significant difference at *p* < 0.05. Limit of Quantification of oxalates: 2.5 mg; Limit of Quantification of saponins: 0.05 mg. As for the rounding method, it was to two decimal places considering its was ≥ 5 or < 5.

Despite the need to increase plant‐food consumption, there have been some concerns raised about whether they are beneficial because of the various antinutrient compounds they contain (Petroski and Minich 2020). Antinutrients can interfere with the bioavailability of essential nutrients (López‐Moreno et al. 2022). Table 4 shows that antinutrient (saponins, oxalates, cyanogenic glycosides, phytates, and tannins) content decreased significantly (*p < 0.05*) in the formulated and the optimal biscuits compared to the initial matrix. For example, taking into account the saponins and tannins content, the values were 0.87 ± 0.21, 0.27 ± 0.10, and 0.53 ± 0.31 mg/100 g for saponins and 618.95 ± 5.10, 389.00 ± 3.02, 529.05 ± 4.20 mg/100 g for tannins respectively for F1, F2 and F3 while these values were 0.18 ± 0.01 mg/100 g (saponins) and 181.11 ± 0.35 mg/100 g (tannins) in OB (optimal biscuit). The levels of antinutrient significantly decreased after processing methods and remained within reported safe limits comparable to the results obtained by Oleghe et al. (2024) for the nutrient and antinutrient of biscuits prepared from fermented and unfermented ternary mixture flours. Their values were between 0.72–1.69 mg/g for tannins while saponins values were between 12.49–23.49 mg/g depending on fermented and unfermented samples. The reduction in antinutrient content in biscuits, unlike in matrices, can be explained by heat treatment, which would have reduced them (Tambo and Natarajan 2025). The levels of these antinutrients in the formulated biscuits did not pose a problem in terms of the bioavailability and digestibility of nutrients, as the quantities were below the critical values (180 mg/100 g for oxalates and 250 mg/100 g for phytates) (Tambo and Natarajan 2025). Furthermore, this suggests the application of treatments such as fermentation, soaking, germination, roasting, charring and boiling in order to reduce antinutrients in the initial matrix and the final product (Kenfack et al. 2021).

### 3.3 Physical and Functional Properties of Plant Materials and Different Biscuits Formulated

Table 5 shows functional and physical properties of tiger nut, dana, avocado, and formulated biscuits. These properties affect the preservation, packaging, and palatability of the final product (Tambo et al. 2019). The bulk density of the biscuits was subdivided into three groups as follows: 0.42–0.46 g/mL (M2, M4, M5, and M9), then 0.48–0.51 g/mL (M1, M3, M6, and M8), 0.52 g/mL for optimal biscuit, and finally 0.56 g/mL for the highest value (M7). Regarding plant materials, the mass density ranged from 0.32 (dana) to 0.67 g/mL (tiger nut). These values were close to those obtained by Dogruer et al. (2023), whose values ranged from 0.48 to 0.78 g/mL when using chickpea, carob, and hazelnut flours in the formulation of biscuits. The values obtained depend on the proportions of tiger nut, which is rich in lipids. The relatively high mass density of the optimal biscuit is thought to be linked to its high lipid and protein content compared to other formulations. Indeed, Tambo et al. (2019) report that foods rich in lipids and carbohydrates have high mass densities due to the high molecular weight of these molecules. The WAC (water absorption capacity) and OAC (oil absorption capacity) of the formulated biscuit decreased compared to that of initial flours. The values were in the range 8%–19.5% and 10%–23% in formulated biscuits, while the values ranged between 0%–26% and 0.20%–20% in raw matrix respectively for WAC and OAC. Dana flour has a higher affinity for water compared to tiger nut flour, given that the values of water absorption capacities were 26% and 16.2%, respectively. It has been reported that the existence of non‐polar side chains, which interact with lipid hydrocarbon chains through hydrophobic interactions, may be responsible for the variation in the oil‐binding capacities of flours. Furthermore, hydrophobic proteins are identified as the primary factor in oil absorption. It has been reported that the existence of non‐polar side chains, which interact with lipid hydrocarbon chains through hydrophobic interactions, may be responsible for the variation in the oil‐binding capacities of flours. Moreover, hydrophobic proteins are identified as the primary factor in oil absorption. Similarly, the presence of various hydrophilic carbohydrates and various protein structures could lead to a different water retention capacity of the flours (Tagodoe and Nip 1994; Du et al. 2014). The difference in protein, fiber, and carbohydrates content of tiger nut and dana in the present study could therefore influence the results obtained concerning WAC and OAC. These two properties influence the acceptability, texture, and flavor of the products. The better oil retention capacity obtained with sample M2 would also explain its good texture (Tambo and Natarajan 2025). pH affects food intake and acceptability, and formulations with pH levels close to neutral are generally the most acceptable. The pH of biscuits was in the range 6.19–6.49. This result was similar to the results obtained by Kumar and Samsher (2016) who obtained pH values ranging from 6.23 to 6.83 in biscuits formulated using multigrain flours during ambient conditions. The SR ranged from 73.46% (Dana) to 29.10% (M6). This parameter was mainly influenced by the proportion of dana flour, which can be due to its high carbohydrate content. Indeed, Tambo et al. (2019) reported a positive correlation between SR and carbohydrate content in the flours of cassava and maize. The OAC/WAC ratio provides information on the affinity for oil or water, and this Table 5 shows that the biscuits currently have a major affinity for oil, with ratios above 1 for most of them. This confirms the oil retention capacity values and can be explained by the high content of hydrophobic amino acids in dana and tiger nut proteins.

**TABLE 5:** Functional and physical properties of tiger nut, dana, avocado, and formulated biscuits.

Samples	WAC (%)	OAC (%)	BD (g/mL)	SR (%)	pH	OAC/WAC
Matrix for biscuit formulation						
F1	16.20 ± 1.50^b^	20.00 ± 0.20^a^	0.67 ± 0.01^a^	62.36 ± 6.70^b^	5.96 ± 0.04^a^	1.32 ± 0.48^a^
F2	26.00 ± 2.82^a^	10.00 ± 2.82^b^	0.32 ± 0.32^b^	73.46 ± 0.69^a^	5.96 ± 0.04^a^	0.38 ± 0.06^b^
F3	0.00 ± 0.00^c^	0.20 ± 0.10^c^	0.10 ± 0.00^c^	0.00 ± 0.00^c^	5.98 ± 0.05^a^	0.00 ± 0.00^c^
Formulated biscuits based on mixture design						
M1	18.00 ± 2.83^ab^	23.00 ± 1.41^a^	0.48 ± 0.01^b^	73.33 ± 1.53^a^	6.41 ± 0.09^abc^	1.28 ± 0.12^bc^
M2	12.80 ± 3.96^bcd^	23.00 ± 4.24^a^	0.46 ± 0.00^c^	54.80 ± 1.12^a^	6.34 ± 0.12^bc^	1.83 ± 0.23^a^
M3	10.00 ± 2.83^cd^	15.00 ± 1.41^bc^	0.51 ± 0.00^b^	53.40 ± 1.20^b^	6.47 ± 0.04^ab^	1.54 ± 0.29^ab^
M4	16.00 ± 0.00^abc^	10.00 ± 2.83^c^	0.49 ± 0.00^c^	51.30 ± 1.99^a^	6.49 ± 0.01^a^	0.62 ± 0.17^d^
M5	14.80 ± 1.13^abc^	11.00 ± 1.41^c^	0.45 ± 0.00^c^	50.70 ± 1.69^a^	6.32 ± 0.02^cd^	0.74 ± 0.15^d^
M6	8.00 ± 0.00^cd^	14.00 ± 2.83^bc^	0.48 ± 0.00^b^	29.10 ± 1.00^b^	6.40 ± 0.02^abc^	1.17 ± 0.35^ab^
M7	14.00 ± 2.83^abcd^	18.00 ± 2.83^ab^	0.56 ± 0.00^a^	39.90 ± 1.46^a^	6.54 ± 0.04^a^	1.29 ± 0.05^bc^
M8	18.00 ± 2.83^ab^	15.00 ± 1.41^bc^	0.49 ± 0.01^b^	57.96 ± 1.31^a^	6.34 ± 0.04^bc^	0.85 ± 0.21^cd^
M9	19.50 ± 4.95^a^	13.00 ± 4.24^bc^	0.42 ± 0.00^c^	65.42 ± 1.59^a^	6.19 ± 0.03^d^	0.66 ± 0.05^d^
Optimal gluten‐free biscuit						
OB	11.00 ± 2.24	14.00 ± 0.02	0.52 ± 0.04	52.36 ± 1.71	6.49 ± 0.01	1.13 ± 0.37

*Note:* F1 = Tiger nut flour, F2 = Dana flour, F3 = Avocado paste, M1‐M9 represent the different biscuits obtained from the three starting matrices based on the mixing plan. OB represents the optimal biscuit. WAC = water absorption capacity, OAC = oil absorption capacity, SR: swelling rate; BD: bulk density. The averages (*n* = 3) in the same column for each parameter that do not share the same letters show a significant difference at *p* < 0.05. As for the rounding method, it was to two decimal places considering it was ≥ 5 or < 5.

### 3.4 Sensory Analysis of the Nine Formulated Biscuits

**FIGURE 1:**
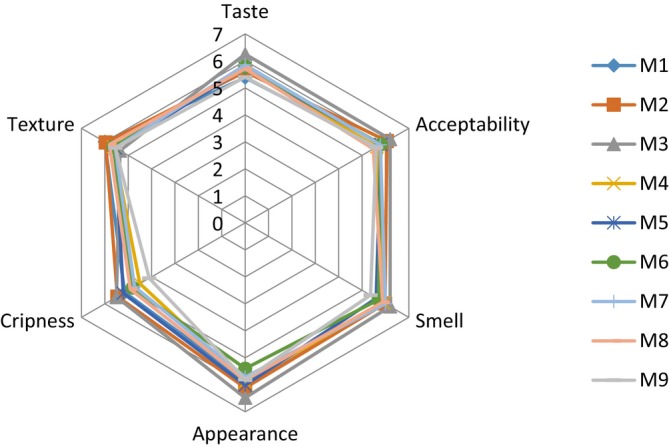
Sensory analysis of the biscuits. M1‐M9 are the different tests (mixture) obtained from the three starting matrices based on the mixture design.

Figure 1 illustrated the sensory profile of the biscuits produced. The overall acceptability of the nine formulations was 5.65, 6.06, 6.2, 5.63, 5.81, 5.86, 5.78, 5.48, and 5.6, respectively, for M1 to M9. On the basis of the fact that the highest value 6.2 was obtained for M3‐formulated biscuit, this formulation can be considered as the most appreciated (aroma, crispness, taste, appearance and overall acceptability) despite the absence of a significant difference between the samples (*p* = 0.27). Biscuit M2 presented the best aroma profile and formulation M9 was the least appreciated biscuit, with an overall acceptability of 5.6. These values were lower compared to those of Njapndounke et al. (2023) who obtained a value of 7.18 as far as the overall acceptability was concerned. Formulation M3 was the most appreciated due to its high lipid and sugar content provided by tiger nuts. The lipids and simple sugars contained in foods improved their organoleptic properties and acceptability. Indeed, Tambo et al. (2024) noted an increased preference for biscuits with a high coconut content. The fact that the sensory attributes of the formulated biscuit were greater than 5 with a global acceptability being 6.2 proves that the product is good and could be acceptable by many consumers (Stone et al. 2012).

### 3.5 Glucose Response and Glycemic Load of Optimal Gluten‐Free Biscuit

**FIGURE 2:**
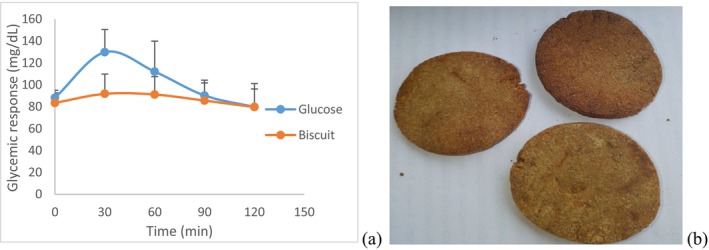
Glycemic response of formulated biscuit: (a) Representation showing glucose response area for optimal gluten‐free biscuit and glucose and (b) optimal gluten‐free biscuit from the mixture of tiger nut, aerial yam, and fat.

To have an idea on the carbohydrate quantity and quality consumed, the measurement of glycemic load has been demonstrated (Emaleku 2017). In this study, as shown in Figure 2, the glycemic index of optimal biscuit was 53.81 ± 5.48 while the glycemic load was 3.4 ± 0.56. Peak glucose values were observed at 30 min for both the glucose reference and the optimal biscuit. Foods have been classified into three categories based on their glycemic index (GI): low (GI ≤ 55), medium (GI: 56–69), and high (GI ≥ 70), according to the work of Vega‐Lopez et al. (2018). Similarly, Gbenga‐Fabusiwa et al. (2019) classified foods into three categories based on glycemic load (GL): low (GL ≤ 10), moderate (11–19), and high (GL ≥ 20). Hence, according to the works of these authors, the obtained optimal biscuit has a low glycemic index and low glycemic load. The fact that the GI and GL of the biscuit was low indicates that the optimal gluten free biscuit made from tiger nut, dana and fat would be a credible alternative in the dietary management of patients with hyperglycemia. This low glycemic index, combined with the low content of reducing sugars, supports the previous assertion and would suggest a low glycemic response. Thomas and Elliott (2010) and Evert et al. (2014) have reported that diets with a low glycemic index and/or low glycemic load improve glycemic control in patients with type 2 diabetes. This could be explained by the nutritional composition of the optimal biscuit, particularly its high fiber content of 18.95%, which means that carbohydrates will be digested fairly slowly. The presence of fiber leads to rapid satiety in consumers due to their strong ability to bind water molecules and swell, and a reduction in digestibility and therefore carbohydrate absorption, which increases the speed of intestinal transit. The obtained GI was in the range of values obtained by Njapndounke et al. (2023) but the GL was not in the range of their values when study with the formulation of gluten‐free biscuit from cowpea and banane cochon flour was performed. It is known that glucose response to a reference food is not constant in the same healthy individual, when measured in a short interval, even being very rigorous with the methodology used (Hirsch et al. 2013), consequently, the values found for glycemic index and glycemic load of optimal biscuit may differ under different circumstances thereby affecting variability and reproducibility. Studies have shown that a person's state of health contributes significantly to the variability in glycemic index (Nirupa et al. 2016). In our study, participants were in good health with no family history of diabetes, and non‐smokers and this would be a good indication that the results are reproducible if the same working conditions are maintained (Figures 1 and 2).

## 4 Conclusions

The formulation of gluten‐free biscuit with high quality in terms of hedonic properties and nutritional value is the greatest challenge. *Dioscorea bulbifera* (Dana), *Cyperus esculentus* (tiger nut), and *Persea americana* (avocado) used in this study have demonstrated their ability in biscuit production by completely replacing wheat flour with flours from these local food plants. The optimal gluten‐free biscuit was obtained using a proportion of 58.40% tiger nut, 25% dana, and 16.59% fat. This optimal biscuit possesses important characteristics in terms of nutritional composition (fiber, ash, lipids, and essential minerals), as well as hedonic and techno‐functional properties. The optimal biscuit also had a low content of antinutrients, particularly cyanide, tannins, and phytates, and a high antioxidant potential. Furthermore, consumption of the optimal biscuit lowered glycemic parameters with a glycemic index and glycemic load of 53.81 and 3.4, respectively. This formulation, while promoting underutilized local plant materials could thus constitute a promising alternative for most gluten‐free foods that are often rich in carbohydrates but lacking other nutrients (protein, lipids, minerals, and fiber) needed for metabolic activities. However, further research could focus on optimizing preservation processes and nutrient stability during storage, clinical trials to validate the effect of this optimal biscuit on glycemic response and other diseases like obesity, and finally investigate how various processing methods could affect the properties of these ingredients. Ultimately, this study opens up promising prospects for the formulation of functional and inclusive bakery products that combine health, sustainability, and the promotion of local resources.

## Author Contributions

**Hilaire Macaire Womeni:** writing – review and editing, validation, methodology, project administration, resources. **Booh'boo Tchassi:** conceptualization, investigation, writing – review and editing, methodology, validation, data curation, formal analysis, software, resources. **Donald Sévérin Dangang Bossi:** writing – review and editing, validation, visualization, methodology, supervision, resources. **Stephano Tambo Tene:** resources, software, methodology, validation, writing – original draft. **Ghislain Maffo Tazoho:** conceptualization, investigation, writing – original draft, methodology, validation, visualization, software, supervision, resources. **Inocent Gouado:** methodology, validation, writing – review and editing, project administration, resources.

## Funding

The authors have nothing to report.

## Ethics Statement

This study was approved by the Regional Ethics Committee for Human Health Research in the West Region of Cameroon (CRERSH‐West, ethical clearance number No. 495/28/05/CE/CRERSH‐OU/VP).

## Consent

Written informed consent was obtained from all study participants.

## Conflicts of Interest

The authors declare no conflicts of interest.

## Data Availability

The data supporting the findings of this study is available from the corresponding author upon reasonable request.
